# Totally Implantable Bidirectional Neural Prostheses: A Flexible Platform for Innovation in Neuromodulation

**DOI:** 10.3389/fnins.2018.00619

**Published:** 2018-09-07

**Authors:** Philip A. Starr

**Affiliations:** Professor of Neurological Surgery, University of California, San Francisco, San Francisco, CA, United States

**Keywords:** neural prostheses, brain-computer interface, deep brain stimulation, bidirectional interface, oscillatory brain activity, electrocorticography, local field potential, brain sensing

## Abstract

Implantable neural prostheses are in widespread use for treating a variety of brain disorders. Until recently, most implantable brain devices have been unidirectional, either delivering neurostimulation without brain sensing, or sensing brain activity to drive external effectors without a stimulation component. Further, many neural interfaces that incorporate a sensing function have relied on hardwired connections, such that subjects are tethered to external computers and cannot move freely. A new generation of neural prostheses has become available, that are both bidirectional (stimulate as well as record brain activity) and totally implantable (no externalized connections). These devices provide an opportunity for discovering the circuit basis for neuropsychiatric disorders, and to prototype personalized neuromodulation therapies that selectively interrupt neural activity underlying specific signs and symptoms.

## Introduction

Implantable bidirectional neural prostheses are devices that combine neurostimulation with neural sensing. These may be implanted in the central or peripheral nervous system. Here, we focus on bidirectional interfaces that are implanted in the central nervous system (CNS). Most implantable CNS prostheses in current clinical use are unidirectional, restricted to either stimulation or sensing only. An example of a stimulation-only application is deep brain stimulation (DBS) for Parkinson's disease (PD) and other movement disorders (Almeida et al., 2017). In this clinical therapy, multipolar leads implanted in the basal ganglia or thalamus are connected to an implanted pulse generator (IPG) that delivers current, and can be controlled externally by radiotelemetry. An example of a sensing-only CNS prosthesis is the “BrainGate” multielectrode cortical implant (Tsu et al., 2015). Implanted in motor cortex in patients with paralysis, BrainGate uses multichannel single unit activity to achieve neural control of external mechanical effectors.

Another important distinction in implantable neural prostheses is between those requiring hardwired connections to communicate with external computers, vs. those that are totally implantable with wireless data transmission. Some devices with a sensing function, such as BrainGate for paralysis, are based on sensing unit activity from a multichannel microelectrode array. Unit activity may be important to achieve the highest number of degrees of freedom for optimal mechanical control. However, the high bandwidth needed for sensing action potentials has necessitated data transmission through hardwired externalized connections. This configuration reduces patient mobility, may be challenging for home use, and elevates infection risk, limiting device lifespan. Totally implantable interfaces, in contrast, are more practical for long term ambulatory use.

Several *bidirectional, totally implantable* neural interfaces designed for CNS use are now available. These include the Responsive Neurostimulation system (RNS) manufactured by Neuropace, Inc. (Morrell, 2011), and Activa PC+S, manufactured by Medtronic, Inc. (Rouse et al., 2011). Their technical capabilities and disease applications are summarized in Table 1. RNS has one indication approved by the United States Food and Drug Adminstration (medically intractable partial epilepsy), while Activa PC+S is investigational only in the United States. An additional device, the Synapse DBS system (Nexeon, Inc.) is available in Europe, but use of its sensing function has not yet been reported. Both RNS and Activa PC+S have the capability to embed feedback control algorithms in the pulse generator, and to stream neural data wirelessly to external computers (Table 1). These capabilities open up many new applications for this type of brain-computer interface, and may transform some of the most common existing applications. While initial therapeutic advances for totally implantable bidirectional interfaces have been in movement disorders and epilepsy, device platforms and conceptual frameworks developed for these conditions may also translate to alleviation of disorders of memory, mood, and executive function. Here, we briefly review technical and conceptual foundations for emerging applications of CNS bidirectional neural interfaces in motor and non-motor systems.

**Table 1:** Comparison of two wireless bidirectional devices designed for brain implantation.

**Device feature**	**Activa PC+S (Medtronic) (Rouse et al., 2011; Swann et al., 2017)**	**Responsive neurostimulator (Neuropace) (Sun et al., 2008; Sun and Morrell, 2014)**
Clinical indications approved by the United States FDA	Investigational only	Epilepsy: adults with partial onset seizures that have not been controlled with two or more antiepileptic medications
lead connectivity	Up to two quadripolar leads	Up to two quadripolar leads
Pulse generator location	Chest	Skull
Rechargeable?	No	No
Designed for continuous stimulation?	Yes	No
Maximum number of channels that can be sensed simultaneously (time series)	Four (Two time series, two spectral power in predefined bands)	Eight
Maximum sampling rate	800 Hz	250 Hz
Data collection modes at home	Time series data or spectral power in predefined bands may be stored on device, triggered by event detection, scheduled time of day, or patient programmer	Time series data can be stored on device, triggered by event detection, responsive stimulation, scheduled time of day, or magnet (patient programmer)
Type of detection algorithm that can be embedded	Classifier based on spectral power in predefined frequency band	Classifier based on predefined time domain and frequency domain tools: area, line-length, and half-wave
Available stimulation parameters	Voltage or current controlled, biphasic square waves, frequency 2–250 Hz, amplitude 0–10.5 V or 25.5 mA, pulse widths 60–450 μs	Current controlled, biphasic square waves, frequency 1 to 333 Hz, amplitude 1–12 mA, pulse widths 40 to 1,000 μs

## Use of field potential recordings in neural interfaces

Both RNS and Activa PC+S are designed for sensing local field potentials (LFPs) recorded from “macroelectrodes,” such as depth electrodes and DBS leads, or electrocorticography (ECoG) potentials recorded from subdural strip leads, rather than single unit activity from microelectrodes. Transmission of field potential data has more modest bandwidth requirements and signal complexity than unit activity, allowing these devices to be totally implanted with wireless connectivity. Field potentials are composed of the summed, synchronized neural activity generated by the population of neurons in close proximity to the recording lead. LFPs and ECoG potentials maybe used to decode critical elements of brain network activity, and are well suited to many BCI applications. In systems neuroscience, it is increasingly recognized that complex brain functions are often encoded in the pattern of synchronization within and between populations of neurons, and that this synchronization is usually oscillatory in nature (Voytek and Knight, 2015; Yuste, 2015). LFP and ECoG potentials are ideal for assessment of oscillatory synchronization. Low frequency (<100 Hz) oscillatory rhythms reflect subthreshold fluctuations in transmembrane potential that are synchronized across a large population of neurons. These fluctuations statistically influence the firing of action potentials. In ECoG recordings, high frequency broadband activity (50–200 Hz), often called “high gamma,” tracks local cortical function (Crone et al., 1998) and provides a surrogate measure of population spiking activity (Manning et al., 2009). Thus, ECoG offers an alternative means to assess population spiking activity that is much more practical than chronic multichannel unit recording.

The importance of oscillatory activity in brain function is underscored by the “communication through coherence” hypothesis (Fries, 2005), positing that functionally connected brain regions communicate by oscillating together in a preferred phase relationship. When regions are phase coherent at an optimal phase lag, action potentials in efferent fibers from one structure will have a greater tendency to produce spiking in its target structure, based on arrival at the correct phase of transmembrane potential oscillations. One implication of this conceptual framework is that analysis of phase coherence between structures in a network may be critical for understanding that network's function. Thus, effective sensing in neuroprosthetics may require multisite recording from multiple leads in a network of related structures, rather than single site recording.

Alterations in oscillatory synchronization are increasingly recognized as fundamental to a variety of brain disorders (Voytek and Knight, 2015). In movement disorders, for example, specific motor signs are correlated with exaggerated network oscillations at characteristic frequencies: 13–30 Hz (beta) rhythms for hypokinetic states such as bradykinesia in PD (Oswal et al., 2013), 60–90 Hz (gamma) for hyperkinetic states (Swann et al., 2016), 4–8 Hz (theta) for dystonic postures (Neumann et al., 2017a), and 10 Hz (twice tremor frequency) for parkinsonian tremor (Timmermann et al., 2003). Derangements of synchronization are also recognized in disorders of mood and cognition (Voytek and Knight, 2015). For example, alpha oscillations in limbic structures correlate with the severity of depression in patients undergoing DBS of the subgenual cingulate gyrus or bed nucleus of the stria terminalis (Neumann et al., 2014). Beta band connectivity between subthalamic nucleus and lateral prefrontal cortex influences decision making, and alterations in this network may underly impaired executive function in PD (Zavala et al., 2017).

## Development of adaptive stimulation algorithms

The identification of oscillatory rhythms underlying specific signs and symptoms of brain disorders raises the possibility of designing tailored neurostimulation therapies that deliver a “desynchronizing” stimulus when neural correlates of the targeted problems are detected by field potential sensing. This therapeutic approach is often referred to as adaptive, feedback controlled, closed loop, or “smart” neurostimulation (Meidahl et al., 2017). Chronic adaptive neurostimulation therapies tailored to specific signs and symptoms can be implemented using the newly available bidirectional interfaces, since they have both the capability for wireless transmission of data to external computers (necessary for personalized signal discovery), and the capability for embedded closed loop control. RNS was the first such system implanted in humans, and remains the only one to have an FDA approved indication in the USA (Table 1). Developed for epilepsy (Morrell, 2011), RNS has also been tested for feedback controlled thalamic stimulation for the suppression of tics in Tourette's syndrome (Molina et al., 2017). RNS has limited applicability to non-paroxysmal disorders (such as Parkinson's disease and mood disorders), since continuous or near-continuous stimulation with this system would result in rapid depletion of its non-rechargeable battery.

**Figure 1:**
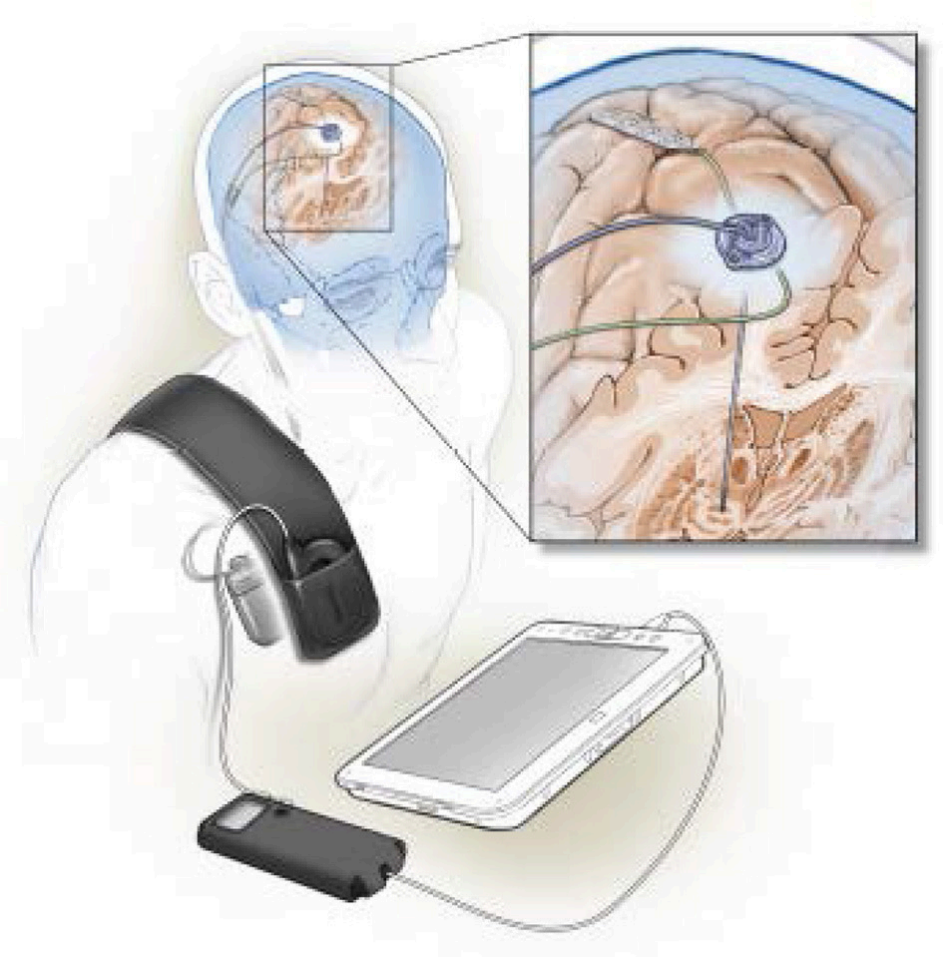
Illustration of a totally implanted bidirectional neural interface designed for chronic multisite recording in humans, as well as therapeutic neurostimulation. Quadripolar electrode arrays are implanted in the subthalamic nucleus and over motor cortex (enlarged view in inset), and attached to Activa PC+S (Medtronic Inc.) implanted over the pectoralis muscle. Data are non-invasively downloaded to an external tablet computer by radiotelemetry. The device is used to characterize networks related to abnormal movement in Parkinson's disease (Swann et al., 2016) and to prototype control algorithms for closed loop stimulation (Swann et al., 2018). This art, by UCSF medical illustrator Ken Probst, is also published in Swann et al. (Swann et al., 2017), with permission.

Activa PC+S (Medtronic) is the first totally implantable bidirectional neural interface designed for continuous use. It became available in the USA in 2013, but only for investigational studies. Early uses of Activa PC+S have focused on signal discovery in movement disorders, including identification of neural correlates of the dyskinetic state (Figure 1; Swann et al., 2016), gait freezing (Syrkin-Nikolau et al., 2017), or disease severity (Neumann et al., 2017b) in PD, and of tics in Tourette's syndrome (Shute et al., 2016). Such personalized neural signatures have been used to prototype adaptive stimulation algorithms in Parkinson's disease (Swann et al., 2018) and essential tremor (Herron et al., 2017). PC+S can be used as a wireless, totally implantable motor cortex prosthesis for paralysis (Vansteensel et al., 2016), allowing practical long term use outside of hospital or laboratory environments, at the expense of much reduced bandwidth compared to externalized systems such as BrainGate (Tsu et al., 2015). Totally implantable neural interfaces also provide the first opportunities for chronic invasive human brain recording in naturalistic environments and in freely moving subjects, and can thus contribute to fundamental studies in systems neuroscience (Aghajan et al., 2017).

Activa PC+S is expected to be replaced in 2018 by a second generation version, Summit RC+S (Medtronic). RC+S is rechargeable, allows more sophisticated embedded closed loop algorithms than its predecessor, can stream data at a distance to external receivers, and may offer improved signal to noise characteristics, with more reliable detection of high gamma (>100 Hz) activity (Swann et al., 2017; Wozny et al., 2017). A recharging capability may also allow more intensive data collection than is possible using Activa PC+S, a primary cell device, since continuous sensing shortens its battery life (Ryapolova-Webb et al., 2014).

## Applications to non-motor systems

Other than for epilepsy, there has been limited therapeutic use of bidirectional neural interfaces in non-motor systems. An early application may be the treatment of non-motor features of Parkinson's disease, in patients already undergoing standard DBS for their motor signs (Deeb et al., 2016). Chronic sensing (Neumann et al., 2014) may also suggest physiological signatures of obsessive-compulsive disorder and major depression (Deeb et al., 2016). Of note, continuous open loop stimulation paradigms showed early successes in mood disorders, but in general have failed to show sufficient efficacy in randomized multicenter trials (Widge et al., 2016). This motivates a new approach in psychiatric neuromodulation, based on discovery of personalized electrophysiologic signatures of specific symptoms, followed by embedding of adaptive algorithms that provide a desynchronizing stimulus only in response to detection of those signals.

Another therapeutic paradigm in cognitive or psychiatric applications could be based on training paradigms that utilize neural data streamed from implantable devices. An operant conditioning approach, in which subjects learn how to voluntarily modulate their own brain rhythms assisted by visualization of data streamed in near real time, has been demonstrated for the motor beta rhythm in PD (Khanna et al., 2017). This general approach could prove especially useful in posttraumatic stress disorder (Langevin et al., 2016), or other disorders in which pathological brain patterns are learned through experience.

## Conclusions

Simple, unidirectional (open loop) totally implanted neural interfaces are now established in the therapy of the most common movement disorders. A new generation of more sophisticated bidirectional, totally implanted CNS prosthetics promises to enhance existing applications and to catalyze the development of novel neurostimulation therapies for disorders of mood and cognition. The major barrier to new indications is the lack of understanding of the neural circuitry underlying psychiatric and cognitive disorders. This knowledge gap is being addressed by chronic human brain recording from bidirectional interfaces, to capture signals that correlate with spontaneous or therapeutically induced fluctuations in symptom severity. This signal discovery phase is expected to lead to personalized circuit-based therapies in which brain sensing is combined with stimulation to target brain rhythms underlying specific cognitive or psychiatric symptoms.

## Author contributions

The author confirms being the sole contributor of this work and approved it for publication.

### Conflict of interest statement

The author declares that he has a US patent related to the methods described in this article.
